# Placental Adaptation to Early-Onset Hypoxic Pregnancy and Mitochondria-Targeted Antioxidant Therapy in a Rodent Model

**DOI:** 10.1016/j.ajpath.2018.07.027

**Published:** 2018-12

**Authors:** Anna M. Nuzzo, Emily J. Camm, Amanda N. Sferruzzi-Perri, Thomas J. Ashmore, Hong-wa Yung, Tereza Cindrova-Davies, Ana-Mishel Spiroski, Megan R. Sutherland, Angela Logan, Shani Austin-Williams, Graham J. Burton, Alessandro Rolfo, Tullia Todros, Michael P. Murphy, Dino A. Giussani

**Affiliations:** ∗ Department of Surgical Sciences, University of Turin, Turin, Italy; † Department of Physiology, Development and Neuroscience, University of Cambridge, Cambridge, United Kingdom; § Medical Research Council Mitochondrial Biology Unit, University of Cambridge, Cambridge, United Kingdom; ‡ Centre for Trophoblast Research, Cambridge, United Kingdom

## Abstract

The placenta responds to adverse environmental conditions by adapting its capacity for substrate transfer to maintain fetal growth and development. Early-onset hypoxia effects on placental morphology and activation of the unfolded protein response (UPR) were determined using an established rat model in which fetal growth restriction is minimized. We further established whether maternal treatment with a mitochondria-targeted antioxidant (MitoQ) confers protection during hypoxic pregnancy. Wistar dams were exposed to normoxia (21% O_2_) or hypoxia (13% to 14% O_2_) from days 6 to 20 of pregnancy with and without MitoQ treatment (500 μmol/L in drinking water). On day 20, animals were euthanized and weighed, and the placentas from male fetuses were processed for stereology to assess morphology. UPR activation in additional cohorts of frozen placentas was determined with Western blot analysis. Neither hypoxic pregnancy nor MitoQ treatment affected fetal growth. Hypoxia increased placental volume and the fetal capillary surface area and induced mitochondrial stress as well as the UPR, as evidenced by glucose-regulated protein 78 and activating transcription factor (ATF) 4 protein up-regulation. MitoQ treatment in hypoxic pregnancy increased placental maternal blood space surface area and volume and prevented the activation of mitochondrial stress and the ATF4 pathway. The data suggest that mitochondria-targeted antioxidants may be beneficial in complicated pregnancy via mechanisms protecting against placental stress and enhancing placental perfusion.

The placenta is the main interface between the mother and fetus, and it regulates intrauterine development by supplying nutrients and oxygen required for fetal growth. There is now clear evidence that the placenta can sense and respond to supply signals arising from the mother and demand signals from the fetus. The organ can adapt morphologically and functionally to these signals (eg, by altering placental and fetal blood flow, fetal nutrient supply, and secretion of signaling molecules, including hormones).^1^ To date, most of the research effort on placental adaptation to adverse pregnancy has focused on maternal nutritional challenges or maternal glucocorticoid overexposure and their effects on placental structure and function.^2^, ^3^ Chronic fetal hypoxia is one of the most common consequences of complicated pregnancy, and it is associated with a variety of maternal, placental, and fetal conditions, including pregnancy at high altitude, gestational diabetes, preeclampsia, and placental insufficiency.^4^, ^5^ Despite this, the effect of hypoxia on the placenta remains relatively unexplored. Decrements in fetal growth have been observed in rodents exposed to hypoxia during mid to late pregnancy.^6^, ^7^, ^8^ Of interest, compared with late-onset hypoxic pregnancy that restricts fetal growth,^8^, ^9^, ^10^ hypoxia exposure earlier in pregnancy does not necessarily reduce fetal or birth weight.^11^, ^12^ This suggests that there are adaptations in maternofetal resource allocation during early-onset hypoxia that help to maintain fetal growth and appropriate development. In relation to the effects of hypoxic pregnancy on placental morphology, the available data from studies in rodents are variable. Increases, decreases, or no difference in placental weights; the surface area and volumes of the maternal and/or fetal compartments; barrier thickness; and transfer of glucose and amino acids and their transporters have been reported.^7^, ^13^, ^14^, ^15^, ^16^, ^17^ This variability is most likely attributable to differences in the duration, severity, and mode of induction, and whether exposure to hypoxia is accompanied by reductions in maternal food intake during the challenge.^9^, ^12^, ^18^, ^19^

Placental oxidative stress is implicated in the pathophysiology of several complications of human pregnancy, including preeclampsia,^20^, ^21^ high-altitude pregnancy,^22^, ^23^ and cases of intrauterine growth restriction.^24^ Closely associated to oxidative stress is disruption of endoplasmic reticulum (ER) function. The ER is a site of integration of various stress responses, including hypoxia, mediated principally through the unfolded protein response (UPR), which aims to restore normal ER function.^25^, ^26^, ^27^ The UPR comprises three highly conserved parallel signaling branches: protein kinase RNA–like ER kinase (PERK), inositol-requiring enzyme, and activating transcription factor (ATF) 6α. Activation of these pathways has been reported in placentas from human intrauterine growth restriction infants with or without preeclampsia^28^, ^29^, ^30^ and, to a lesser extent, in healthy pregnancies at high altitude.^23^

Recently, the potential use of antioxidant therapies to protect the placenta and fetus against oxidative stress in complications of pregnancy and birth has attracted much attention. We developed a rodent animal model of hypoxic pregnancy that minimizes effects on maternal food intake, thereby helping to isolate the effects of hypoxia on the placenta and offspring.^11^, ^31^ Using this model, we have shown that early-onset hypoxia from days 6 to 20 of gestation increases placental size and induces placental oxidative stress and that maternal treatment with the antioxidant vitamin C is protective.^11^, ^31^, ^32^ Although these data provide proof of principle that maternal antioxidant therapy may confer protection to the placenta and offspring in hypoxic pregnancy, in these studies, only high doses of vitamin C were effective. In addition, clinical trials have reported that maternal treatment with vitamin C in human pregnancy complicated by preeclampsia did not prove protective to the mother or baby.^33^, ^34^ Therefore, there is increasing interest in alternative maternal antioxidant therapies to protect the placenta and offspring in complicated pregnancy, with greater translational capacity to the human clinical situation.

Mitochondria-targeted antioxidants might offer a plausible alternative, because most endogenous reactive oxygen species are generated within mitochondria.^35^ The most extensively studied compound of this class is the mitochondria-targeted ubiquinone derivative MitoQ, which can pass easily through all biological membranes and accumulate several hundredfold within mitochondria, thereby enhancing protection from oxidative damage.^36^, ^37^ The use of MitoQ *in vivo* in several different rodent models of human pathology has shown that MitoQ can protect against oxidative damage in adult offspring.^38^, ^39^, ^40^, ^41^, ^42^, ^43^, ^44^, ^45^ Furthermore, long-term oral administration is safe, and unlike other conventional antioxidants, MitoQ does not demonstrate pro-oxidant activity at high doses *in vivo*.^46^, ^47^ An oral preparation of MitoQ has already safely undergone phase 1 and 2 human clinical trials. A study demonstrated that MitoQ can be safely administered for 1 year and is well tolerated by patients.^48^ To date, only one study has investigated the antioxidant benefits of MitoQ in pregnancy, reporting that treatment of the pregnant rat with nanoparticle-bound MitoQ during hypoxic pregnancy could protect fetal brain development.^49^ Therefore, the aim of this study was to investigate the effects of hypoxic pregnancy, with and without maternal treatment with MitoQ, on placental morphologic capacity for substrate transport and to determine whether UPR-sensing mechanisms were affected.

## Materials and Methods

### Experimental Design

All procedures described were approved by the Ethical Review Committee of the University of Cambridge (Cambridge, UK) and were in accordance with UK Animals (Scientific Procedures) Act 1986. Power calculations derived from previously published data using a similar experimental design^11^, ^31^, ^50^ were used to determine the minimum numbers required for statistically valid results, taking into account sex of the offspring and variations in litter size. Virgin Wistar rats (Charles River, Margate, UK; 10 to 12 weeks of age) were mated with male Wistar rats (minimum, 12 weeks of age) overnight. Pregnancy was confirmed by the presence of a copulatory plug (day 0, term approximately 22 days). Pregnant dams were then housed individually (21°C, 60% humidity, 12:12-hour light-dark cycle) with free access to food (Special Diet Services, Witham, UK) and water. Maternal weight and food and water consumption were monitored daily throughout gestation. On day 6 of pregnancy, rats were randomly assigned to either normoxic (21% O_2_) or hypoxic (13% to 14% O_2_) conditions. Two additional normoxic and hypoxic groups were examined and were given the mitochondria-targeted antioxidant MitoQ (500 μmol/L in maternal drinking water), which was prepared fresh daily. Pregnant dams subjected to hypoxia were placed inside a chamber, which combined a polyvinyl chloride isolator with a nitrogen generator, as previously described.^31^, ^32^, ^51^ The experimental design, therefore, consisted of four groups: normoxia (N; *n* = 16 litters), hypoxia (H; *n* = 16 litters), hypoxia with MitoQ (HM; *n* = 18 litters), and normoxia with MitoQ supplementation (NM; *n* = 16 litters). The dose of MitoQ was derived from previous animal studies^39^, ^46^, ^47^, ^52^ and corresponds to an oral dose of approximately 0.05 mg/day per gram in rats.^38^

### Tissue Collection

On day 20 of gestation, all dams underwent euthanasia by carbon dioxide inhalation and cervical dislocation. A maternal blood sample for measurement of hematocrit was taken by cardiac puncture. The pregnant uterus was exposed via a midline incision, and the pups were euthanized via spinal transection. Maternal blood was centrifuged for determination of hematocrit. All fetuses and their associated placentas were weighed. To control for within-litter variation, one placenta was randomly selected and processed for stereology. Another two placentas from each litter were collected and immediately frozen in liquid nitrogen for MitoQ uptake and protein isolation analyses, respectively. Therefore, only one placenta per litter was used for each outcome measure. Only placentas from male pups were collected, to control for sex variation.

### MitoQ Uptake

The uptake of MitoQ was assessed in the placenta, maternal liver, and fetal liver. MitoQ was measured using a liquid chromatography–tandem mass spectrometry assay.^46^ Frozen tissues were homogenized in tris buffer (pH 7.0), extracted with acetonitrile (Sigma-Aldrich, Gillingham, UK), and dried overnight under a vacuum. The extracts were reconstituted, and the MitoQ content was measured using mass spectrometry. Data were analyzed using MassLynx MS software version 4.1 (Waters, Elstree, UK) and expressed relative to a deuterated internal standard. Control samples were spiked with known amounts of MitoQ from 1 to 500 pmol to generate a standard curve; the assay could detect as low as 0.1 pmol MitoQ/100 mg of tissue.

### Placental Histology and Stereology

At post-mortem, the placentas randomly selected for stereology were transversally cut into two halves. One half was immersion fixed in 4% paraformaldehyde, embedded in paraffin wax, and then completely divided into sections (7 μm thick) perpendicular to the chorionic plate (Leica RM 2235 microtome; Leica Microsystems, Wetzlar, Germany). Systematic random sampling was used to select, without bias, 10 sections for analysis.^53^ Hematoxylin and eosin staining of these sections was used to visualize the gross structure of the rat placenta. Immunohistochemistry was performed on sections near the placental midline for markers of mitochondrial stress [glucose-regulated protein 75 (GRP75) and tumorous imaginal disc 1 (TID-1)] and to localize ATF4 and glucose-regulated protein 78 (GRP78). The other half of the placenta was fixed with 4% glutaraldehyde and embedded in Spurr epoxy resin (Taab, Aldermaston, UK). A section (1 μm thick) was cut near to the placental midline and stained with toluidine blue to visualize the structure of the labyrinthine zone.^54^

The Computer Assisted Stereology Toolbox 2.0 system from Olympus (Ballerup, Denmark), fitted with a motorized specimen stage, was used to perform all stereological measurements. All quantitative analyses were performed with the observer (A.M.N.) blinded to the treatment group. To determine the absolute volume of the placenta, a point grid was superimposed on vertically orientated hematoxylin and eosin–stained paraffin sections viewed using a 1.25× objective lens. Points falling on the sample were counted, and the Cavalieri principle was applied to reach a volume estimate^55^: *V*_(obj)_ = *t* × Σ *a* = *t* × *a*_(p)_ × Σ *P*, where *V*_(obj)_ is the estimated placental volume, t is the total thickness of the placenta (total number of sections multiplied by section thickness), *a*_(p)_ is the area associated with each point, and Σ *P* is the sum of points on sections. At ×10 magnification, meander sampling and point counting were used to estimate compartment densities of the three placental zones: labyrinthine zone (LZ), junctional zone (JZ), and decidua basalis: *V*v(struct,ref) = *P*(struct)/*P*(total), where *V*v(struct,ref) is the volume fraction of a compartment (eg, LZ) within a reference space (eg, placenta), *P*(struct) is the number of points falling on the compartment, and *P*(total) is the total number of points falling on the reference space (including the component). The volume densities obtained were converted to absolute quantities by multiplying by total placental volume.^55^, ^56^

Resin sections were used to resolve the labyrinth structure in detail. A 100× objective lens was used, and fields of view within the LZ were selected by meander sampling to determine volume densities, surface densities, and interhaemal membrane thickness. Volume densities of the maternal blood space and fetal capillaries were obtained using a point grid.^54^ Volume densities were converted to absolute component volumes by multiplying by the volume of the LZ. Vascular surface densities for the maternal blood space and fetal capillaries were obtained using a grid formed of cycloid arcs placed over each field of view, and intercepts between maternal blood space boundary and fetal capillary boundary were counted. The following equation was used to determine surface areas: *S*(struct) = [2 × Σ *I*(struct)/*I*(p) × Σ *P*(ref)] × *V*(ref), where Σ *I*(struct) is the total number of intersections of the cycloid arcs with the structure, *V*(ref) is the reference volume, Σ *P*(ref) is the total number of points that hit the reference space, and *I*(p) is the length of the test line associated with each point in the grid.^57^ All surface area densities were converted to absolute surface areas by multiplying by the volume of LZ. Thickness of the interhaemal membrane of the LZ was obtained with a line grid to establish random start points for measuring distances between fetal capillaries and the closest maternal blood space by the method of orthogonal intercepts.^58^ Intercept lengths were multiplied by the factor (8/3)π to correct for plane of sectioning,^59^ and the harmonic mean thickness of the membrane was calculated as the reciprocal of the mean of the reciprocals of the corrected intercept distances. The theoretical diffusion capacity for the interhaemal membrane was calculated using the following equation: *Dvm* = *K* × (mean surface area/mean thickness), where *Dvm* is the diffusing capacity across the LZ membrane, *K* is the Krogh diffusion coefficient for oxygen (17.3 × 10^−8^ cm^2^/minute kPa),^60^ mean surface area is the mean of fetal and maternal surface areas of the interhaemal membrane, and mean thickness is the harmonic mean thickness of the interhaemal membrane. The specific diffusion capacity is an estimate of the diffusing capacity for oxygen in terms of fetal requirements, obtained by expressing *Dvm* per mg of fetal weight.

### Immunohistochemistry

Sections near the placental midline were dewaxed and then rehydrated in water for 10 minutes, incubated with 3% H_2_O_2_ for 15 minutes, and washed in tap water before antigen retrieval was performed (tris-EDTA buffer, pH 9.0; Sigma-Aldrich). Sections were washed with tris-buffered saline (TBS) with 1% Triton-X and 1% Tween-20 (all Sigma-Aldrich) for 30 minutes and then specific binding was blocked with 5% bovine serum albumin in TBS (Sigma-Aldrich) for 1 hour. Sections were then incubated overnight at 4°C with the following primary antibodies: UPR-related proteins anti-GRP78 (1:1000; Transduction Laboratories, BD Biosciences, Oxford, UK) and anti-ATF4 (1:250; Santa Cruz Biotechnology, Wembley, UK). They were also incubated with markers of the mitochondrial matrix: anti–TID-1 (1:100; GeneTex, Wembley, UK) and anti-GRP75 (1:100; Abcam, Cambridge, UK). Negative control samples were obtained by omitting the primary antibody. The following day, sections were washed 15 minutes in TBS with 1% Triton-X and 1% Tween-20, incubated for 1 hour with secondary antibody (Vector Laboratories, Peterborough, UK), and then washed for 15 minutes in TBS with 1% Triton-X and 1% Tween-20. Sections were incubated for 45 minutes in avidin/biotin (Vector Laboratories) in TBS, then washed in TBS for 10 minutes. Staining was visualized with 3,3′-diaminobenzidine tetrahydrochloride/hydrogen peroxide (Sigma-Aldrich) for 2 minutes. Slides were rinsed with water, dehydrated, and then coverslipped with DPX (Sigma-Aldrich).

### OD

The OD of GRP75 and TID-1 immunostaining was measured in the LZ and JZ using a calibrated OD step tablet (ImageJ software version 1.80; NIH, Bethesda, MD; *http://imagej.nih.gov/ij*). For each placenta, 10 fields within each region (LZ and JZ) were examined.

### Western Blot Analysis

Whole placental tissue was homogenized in cell lysis buffer and a mini protease inhibitor cocktail (Roche Diagnostics, East Sussex, UK). The protein concentration of the lysates was measured by a bicinchoninic acid protein assay (Sigma-Aldrich). The samples were mixed with SDS-PAGE gel loading buffer (50 mmol/L tris-HCl, pH 6.8, 100 mmol/L dithiothreitol, 2% SDS, 10% glycerol, and bromophenol blue) and boiled for 5 minutes. Equivalent amounts of protein (1 μg/μL) were resolved by SDS-PAGE, blotted onto nitrocellulose membranes (0.2 μm thick), and probed overnight at 4°C with the following primary antibodies: anti-GRP78 (Transduction Laboratories, BD Biosciences), anti-AKT (Cell Signaling Technology, Hitchin, UK), anti–ATF-4 (Santa Cruz Biotechnology), anti–phosphorylated AKT (Thr308) (Santa Cruz Biotechnology), anti–4-hydroxynonenal (Merck Millipore, Watford, UK), and anti–70-kDa heat shock protein (Enzo Life Sciences, Exeter, UK). Anti–β-actin (Sigma-Aldrich) was used to normalize protein levels. Some membranes were reprobed with antibodies of different molecular weight or those that were raised in a different species. The membranes were analyzed by enhanced chemiluminescence (Amersham Biosciences, Little Chalfont, UK) using Kodak X-OMAT androgen receptor film (Sigma-Aldrich). Films were scanned using a flat-bed scanner (Canon 8000F; Reigate, Surrey, UK), and the intensity of the bands was determined from two or three different exposures (within the linear detection range) using ImageJ analysis software.^61^

### Statistical Analysis

All data are expressed as means ± SEM. Maternal pregnancy variables and biometry, placenta stereology, and molecular analyses were compared statistically using a general linear model test with repeated measures when appropriate (SPSS V24.0; IBM, Armonk, NY). Fetal biometry was assessed using the linear mixed models (SPSS V24.0), which nests offspring data within a maternal identifier, thereby accounting for the shared maternal environment.^62^ For all comparisons, significance was accepted when *P* < 0.05.

## Results

### Maternal and Fetal Biometry

**Figure 1:**
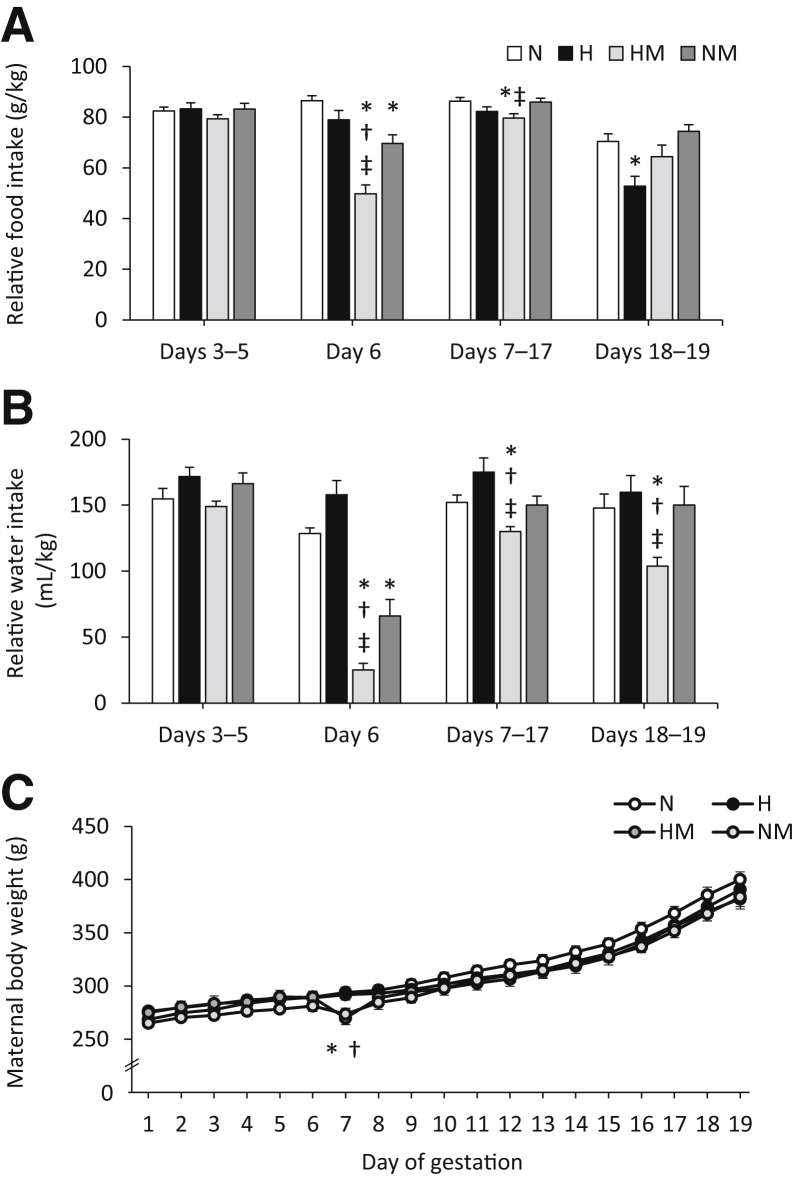
Effects of maternal hypoxia with or without MitoQ treatment on maternal parameters during days 6 to 20 of gestation. **A:** Maternal food intake expressed relative to body weight. **B:** Maternal water intake expressed relative to body weight. **C:** Maternal body weight in normoxic (N), hypoxic (H), hypoxic + MitoQ (HM), and normoxic + MitoQ (NM) pregnancies. Data are expressed as means ± SEM. ^∗^*P* < 0.05 versus N; ^†^*P* < 0.05 versus H; ^‡^*P* < 0.05 versus NM (general linear model repeated-measures test).

Maternal hypoxia induced a significant increase in maternal hematocrit (*P* = 0.002) (Table 1) and placental weight (*P* = 0.002) (Table 2). Body weight and other fetal biometric variables were unaltered by hypoxic pregnancy or MitoQ treatment (Table 2). Similarly, litter size (N, 15.3 ± 0.8; H, 16.8 ± 0.6; HM, 14.5 ± 1.0; NM, 14.00 ± 1.2) and sex ratio (percentage of males: N, 49.5% ± 4.0%; H, 50.6% ± 3.4%; HM, 54.5% ± 4.9%; NM, 46.1% ± 3.8%) were unchanged. Maternal exposure to hypoxia did not alter maternal weight gain with advancing gestation nor reduce maternal food or water intake until days 18 of gestation (Figure 1). Between days 18 and 19 of gestation, all pregnant dams showed a reduction in maternal food intake relative to days 7 to 17 of gestation (all *P* < 0.05), which was more pronounced in hypoxic relative to normoxic pregnancy (*P* = 0.002) (Figure 1B). Maternal treatment with MitoQ in normoxic and hypoxic pregnancy led to a transient, but significant, fall of similar magnitude in maternal food and water intake (Figure 1, A and B) and maternal body weight (Figure 1C) soon after the onset of administration on day 6 of gestation (all *P* ≤ 0.001). Shortly afterward, maternal body weight gain and food and water intake recovered toward control values with advancing gestation in normoxic and hypoxic pregnancy treated with MitoQ. However, in MitoQ-treated pregnancies, water, rather than food, intake appeared more affected (Figure 1).

**Table 1:** Maternal Biometric Data

Variable	N	H	HM	NM
Hematocrit (%)	34.7 ± 1.9	40.9 ± 1.5∗	39.9 ± 1.2∗	37.9 ± 0.6
BW (g)	410.2 ± 7.2	395.6 ± 8.6	387.4 ± 10.4	391.4 ± 8.7
CRL (mm)	190.7 ± 2.0	187.2 ± 3.0	199.6 ± 6.1	184.5 ± 4.1
HD (mm)	22.8 ± 0.5	22.7 ± 0.5	22.5 ± 0.3	23.2 ± 0.3
BMI (kg/m^2^)	11.3 ± 0.3	11.4 ± 0.5	10.0 ± 0.6	11.6 ± 0.5
HD/BW ratio	0.056 ± 0.001	0.058 ± 0.002	0.059 ± 0.002	0.059 ± 0.002

Hematocrit, BW, CRL, HD, BMI, and HD/BW ratio from N, H, HM, and NM dams at day 20 of gestation. Values are means ± SEM.

BMI, body mass index; BW, body weight; CRL, crown-rump length; H, hypoxic; HD, head diameter; HM, hypoxic + MitoQ; N, normoxic; NM, normoxic + MitoQ.

∗ Significant main effect of hypoxia on hematocrit (*P* < 0.05, general linear model test). Number of dams for hematocrit: N = 16; H = 16; HM = 18; and NM = 16. Number of dams for remaining variables: N = 10; H = 10; HM = 11; and NM = 11.

**Table 2:** Fetal Biometric Data

Variable	N	H	HM	NM
BW (g)	3.63 ± 0.05	3.41 ± 0.03	3.72 ± 0.06	3.39 ± 0.04
PW (g)	0.55 ± 0.01	0.62 ± 0.01∗	0.60 ± 0.01∗	0.54 ± 0.01
BW/PW ratio	6.69 ± 0.15	5.77 ± 0.15	6.44 ± 0.18	6.49 ± 0.15
CRL (mm)	33.19 ± 0.30	32.23 ± 0.30	33.12 ± 0.24	32.35 ± 0.31
HD (mm)	7.73 ± 0.07	7.72 ± 0.06	7.78 ± 0.07	7.60 ± 0.06
BMI (kg/m^2^)	3.33 ± 0.04	3.38 ± 0.08	3.43 ± 0.04	3.32 ± 0.05
HD/BW ratio	2.14 ± 0.03	2.25 ± 0.03	2.09 ± 0.04	2.25 ± 0.03

BW, PW, placental efficiency (BW/PW ratio), CRL, HD, BMI, and HD/BW ratio from male fetuses only from N, H, HM, and NM pregnancy at day 20 of gestation. Values are means ± SEM.

BMI, body mass index; BW, body weight; CRL, crown-rump length; H, hypoxic; HD, head diameter; HM, hypoxic + MitoQ; N, normoxic; NM, normoxic + MitoQ; PW, placental weight.

∗ Significant main effect of hypoxia on placental weight (*P* < 0.05, mixed linear model test). Number of fetuses for BW: N = 74; H = 86; HM = 84; and NM = 65. Number of fetuses for remaining variables: N = 59; H = 59; HM = 62; and NM = 54.

### MitoQ Uptake

MitoQ uptake (pmol MitoQ/g wet weight of tissue), measured by a liquid chromatography–tandem mass spectrometry assay, was expressed relative to untreated normoxic and hypoxic dams and their fetuses. By day 20 of gestation, MitoQ accumulation was greatest in the maternal liver [HM, 173 ± 37 pmol/g (*n* = 9); NM, 192 ± 40 pmol/g (*n* = 10)], followed by the placenta [HM, 132 ± 28 pmol/g (*n* = 10); NM, 78 ± 24 pmol/g (*n* = 11)] and then fetal liver [HM, 8.5 ± 2.2 pmol/g (*n* = 10); NM, 11.4 ± 3.7 pmol/g (*n* = 10)].

### Placental Morphology

**Figure 2:**
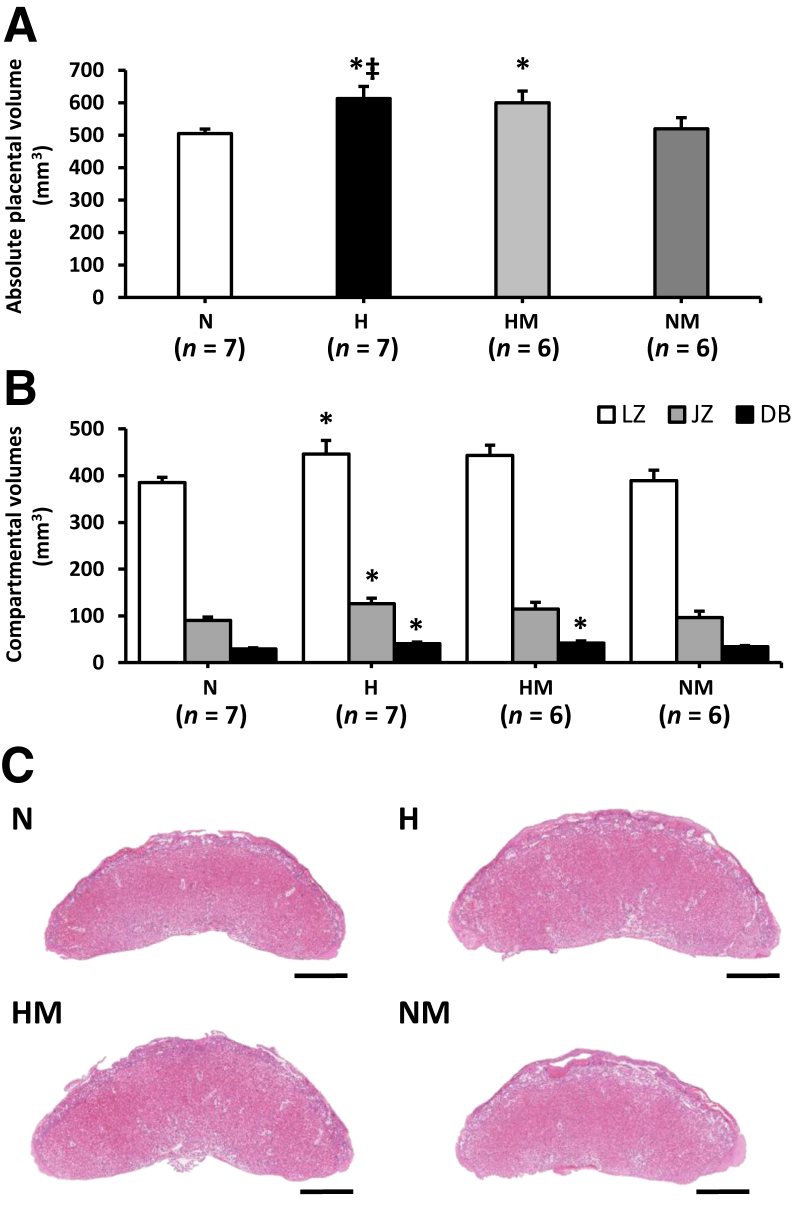
Effects of maternal hypoxia with or without MitoQ treatment on placental volumes at day 20 of gestation. **A** and **B:** Total placental volume (**A**) and compartmental volume (**B**) in normoxic (N), hypoxic (H), hypoxic + MitoQ (HM), and normoxic + MitoQ (NM) pregnancies. **C:** A representative hematoxylin and eosin–stained paraffin section of the placenta is shown for each group. Data are expressed as means ± SEM. ^∗^*P* < 0.05 versus N; ^‡^*P* < 0.05 versus NM (general linear model test). Scale bar = 1 mm (**C**). DB, decidua basalis; JZ, junctional zone; LZ, labyrinthine zone.

**Figure 3:**
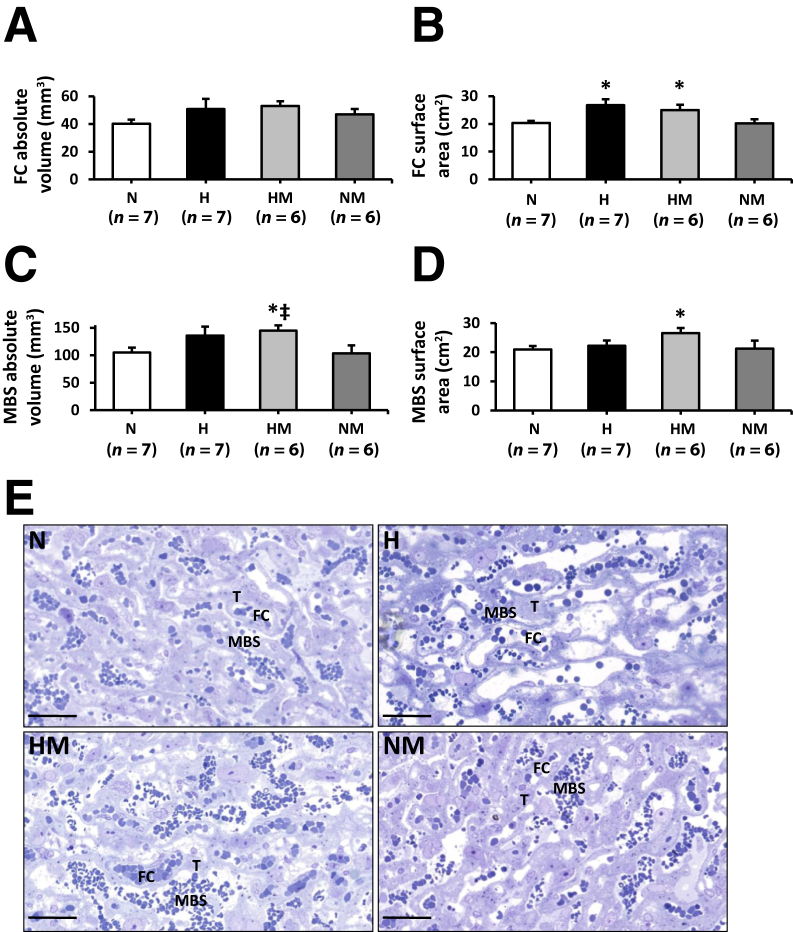
Effects of maternal hypoxia with or without maternal MitoQ treatment on the volume and surface area of fetal capillaries (FCs) and maternal blood spaces (MBSs) at day 20 of gestation. **A–D:** FC absolute volume and surface area (**A** and **B**, respectively) and MBS absolute volume and surface area (**C** and **D**, respectively) in normoxic (N), hypoxic (H), hypoxic + MitoQ (HM), and normoxic + MitoQ (NM) pregnancies. **E:** A representative toluidine blue–stained resin section of the labyrinthine zone is shown from one placenta per group. Data are expressed as means ± SEM. ^∗^*P* < 0.05 versus N; ^‡^*P* < 0.05 versus NM (general linear model test). Scale bars = 50 μm (**E**). T, trophoblast.

**Figure 4:**
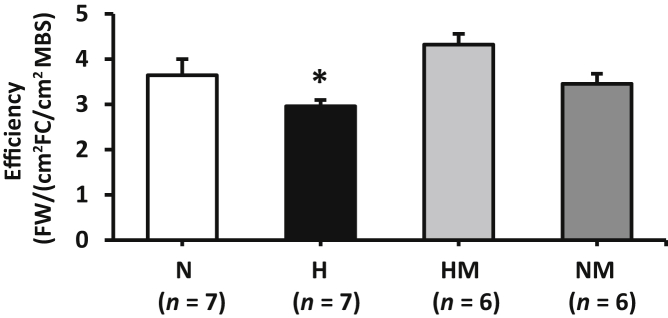
Effects of maternal hypoxia with or without maternal MitoQ treatment on placental efficiency at day 20 of gestation. Fetal body weight (FW) expressed relative to fetal capillary (FC) and maternal blood space (MBS) areas in normoxic (N), hypoxic (H), hypoxic + MitoQ (HM), and normoxic + MitoQ (NM) pregnancies. Data are expressed as means ± SEM. ^∗^*P* < 0.05 versus N (general linear model test).

**Figure 5:**
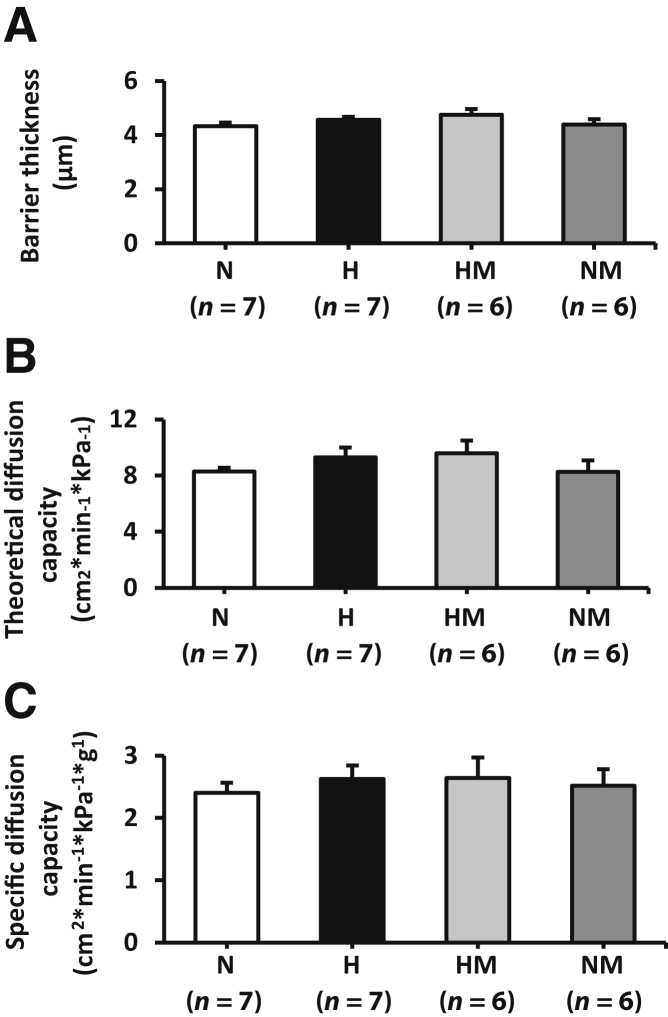
Effects of maternal hypoxia with or without maternal MitoQ treatment on barrier thickness, theoretical diffusion capacity (TDC), and specific diffusion capacity (SDC) of the placental interhaemal membrane at day 20 of gestation. Barrier thickness (**A**), TDC (**B**), and SDC (**C**) in normoxic (N), hypoxic (H), hypoxic + MitoQ (HM), and normoxic + MitoQ (NM) pregnancies. Data are expressed as means ± SEM (**A–C**).

At day 20 of gestation, the absolute volume of hypoxic placentas was greater than that of normoxic placentas (*P* = 0.014) (Figure 2, A and C). The absolute volumes of the labyrinthine zone, junctional zone, and decidua were proportionally increased in hypoxic pregnancies (LZ, *P* = 0.046; JZ, *P* = 0.034; decidua basalis, *P* = 0.015) (Figure 2B). Although hypoxia did not affect total fetal capillary volume in the labyrinthine zone (Figure 3A), total fetal capillary surface area was significantly increased compared with normoxic placentas (*P* = 0.005) (Figure 3B); maternal blood space volume and surface area were unchanged (Figure 3D). Placental efficiency, expressed as the ratio of fetal body weight/fetal capillary area and maternal blood space area, was significantly reduced in placentas from hypoxic pregnancy (*P* = 0.021) (Figure 4). Interhaemal membrane thickness and theoretical and specific diffusion capacity were unaltered in hypoxic pregnancy (Figure 5).

In hypoxic pregnancy treated with MitoQ, absolute placenta volume was increased relative to normoxic pregnancy (*P* = 0.039) (Figure 2, A and C). Furthermore, the absolute volume of the decidua basalis was increased (*P* = 0.010) (Figure 2B). MitoQ treatment in hypoxic pregnancy did not alter absolute fetal capillary volume (Figure 3, A and E); however, fetal capillary surface area was increased relative to placentas from normoxic pregnancy (*P* = 0.049) (Figure 3, B and E). In addition, MitoQ treatment in hypoxic pregnancy increased both maternal blood space volume (*P* = 0.033) (Figure 3, C and E) and surface area (*P =* 0.041) (Figure 3, D and E). Placental efficiency (Figure 4), the thicknesses of the interhaemal membrane, and the theoretical and specific diffusion capacities remained unaltered (Figure 5). In normoxic pregnancy, MitoQ administration did not affect placental morphology (Figures 2, 3, 4, and 5).

### Placental Unfolded Protein Response, Cell Proliferation, and Oxidative and Mitochondrial Stress Signaling Pathways

**Figure 6:**
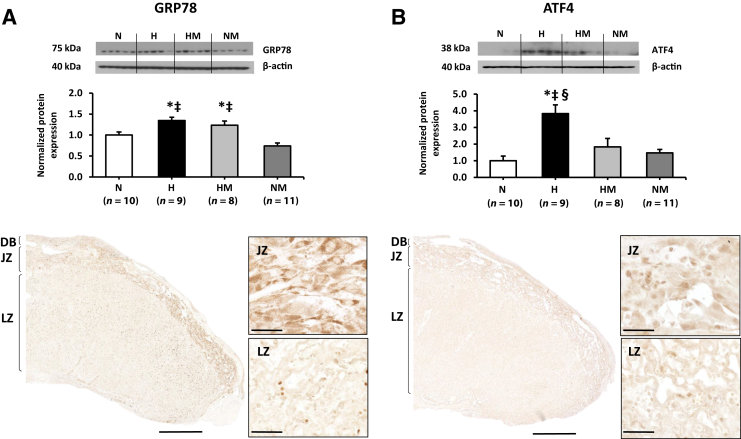
Effects of maternal hypoxia with or without maternal MitoQ treatment on endoplasmic reticulum stress signaling pathway at day 20 of gestation. Representative Western blots and mean densitometry for glucose-regulated protein 78 (GRP78; **A**) and activating transcription factor 4 (ATF4; **B**) in normoxic (N), hypoxic (H), hypoxic + MitoQ (HM), and normoxic + MitoQ (NM) placentas. After normalization to β-actin, the mean density of the samples was expressed relative to normoxic placentas, assigned an arbitrary value of 1. Representative sections show the localization of GRP78 and ATF4 in the labyrinthine and junctional zones of the placenta. Data are expressed as means ± SEM. ^∗^*P* < 0.05 versus N; ^‡^*P* < 0.05 versus NM; ^§^*P* < 0.05 versus HM (general linear model test). Scale bars: 1 mm (**A** and **B**, **bottom left panels**); 50 μm (**A** and **B**, **bottom right panels**). DB, decidua basalis; JZ, junctional zone; LZ, labyrinthine zone.

**Figure 7:**
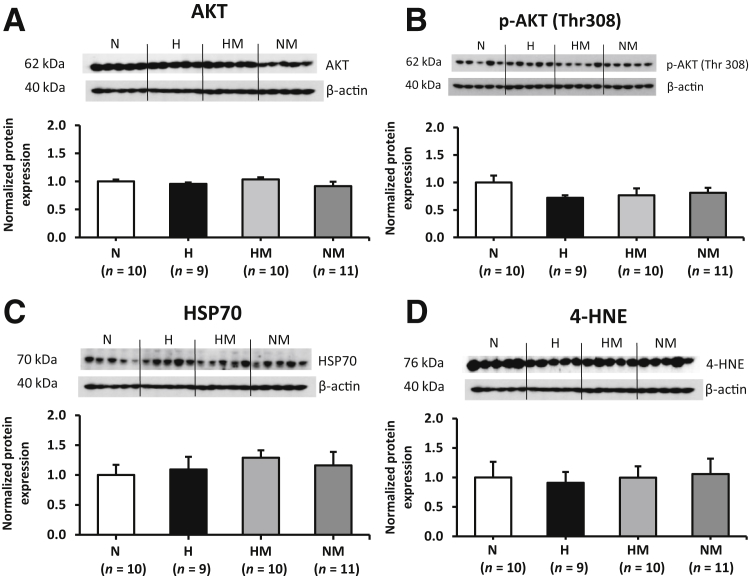
Effects of maternal hypoxia with or without maternal MitoQ treatment on oxidative stress and lipid-peroxidation markers at day 20 of gestation. Representative Western blot analysis and mean densitometry for AKT (**A**), AKT phosphorylation at Thr308 residues (p-AKT Thr308; **B**), 70-kDa heat shock protein (HSP70; **C**), and 4-hydroxynonenal (4-HNE; **D**) in normoxic (N), hypoxic (H), hypoxic + MitoQ (HM), and normoxic + MitoQ (NM) placentas. After normalization to β-actin, the mean density of the samples was expressed relative to normoxic placentas, assigned an arbitrary value of 1. Data are expressed as means ± SEM.

In hypoxic pregnancy, GRP78 (*P* = 0.001) (Figure 6A) and ATF4 abundance (*P* < 0.001) (Figure 6B) were significantly increased in the placenta relative to normoxic pregnancy. In hypoxic pregnancy treated with MitoQ, GRP78 remained elevated relative to normoxic pregnancies (*P* = 0.032) (Figure 6A); however, ATF4 expression was restored to normoxic levels (*P* = 0.130) (Figure 6B). There was no effect of MitoQ supplementation in normoxic pregnancy on GRP78 or ATF4 (Figure 6). Across all treatment groups, GRP78 expression was localized to the JZ, whereas AFT4 staining was seen in both the LZ and JZ (Figure 6). Total AKT (Figure 7A), phosphorylated AKT (Thr308) (Figure 7B), 70-kDa heat shock protein (Figure 7C), and 4-hydroxynonenal (Figure 7D) were unaltered by hypoxia and/or MitoQ.

**Figure 8:**
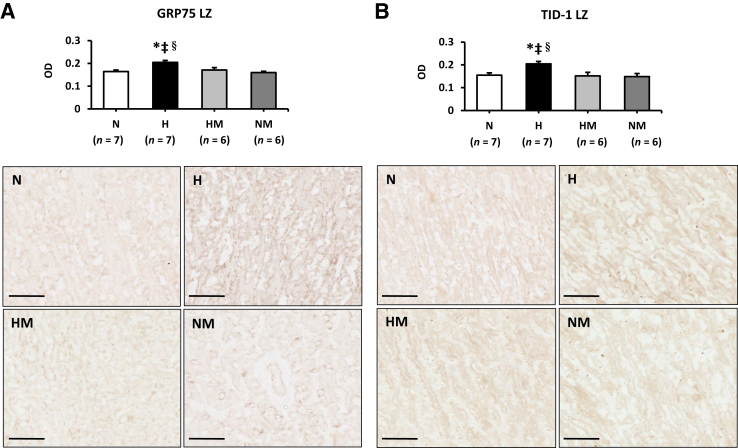
Effects of maternal hypoxia with or without maternal MitoQ treatment on mitochondrial stress at day 20 of gestation. The mean OD of glucose-regulated protein 75 (GRP75; **A**) and tumorous imaginal disc 1 (TID-1; **B**) staining in normoxic (N), hypoxic (H), hypoxic + MitoQ (HM), and normoxic + MitoQ (NM) placentas. Representative sections showing the intensity of GRP75 and TID-1 staining in the labyrinthine zone (LZ) of the placenta. Data are expressed as means ± SEM. ^∗^*P* < 0.05 versus N; ^‡^*P* < 0.05 versus NM; ^§^*P* < 0.05 versus HM (general linear model test). Scale bar = 100 μm (**A** and **B**).

Both GRP75 and TID-1, which localize to the mitochondrial matrix, were ubiquitously expressed throughout the placenta. The staining intensity (OD) of GRP75 was increased in both the LZ (Figure 8A) and JZ [N, 0.23 ± 0.1; H, 0.29 ± 0.02; HM, 0.24 ± 0.01; NM, 0.21 ± 0.01 (both *P* < 0.05)] in hypoxic placentas, but restored with MitoQ treatment. A similar trend was observed with TID-1, which was increased in the LZ in hypoxic pregnancy only (Figure 8B). No changes in TID-1 staining were observed in the JZ for OD (N, 0.18 ± 0.1; H, 0.20 ± 0.01; HM, 0.16 ± 0.02; NM, 0.16 ± 0.01). There was no effect of MitoQ supplementation in normoxic pregnancy on GRP75 or TID-1 staining (Figure 8).

## Discussion

The data show that early-onset hypoxic pregnancy modifies the placental morphologic phenotype that offsets increased signaling in placental UPR pathways to maintain fetal growth. Hypoxic pregnancy increased placental volume and the fetal capillary surface area within the labyrinthine transport zone and induced the UPR and mitochondrial stress, as evidenced by up-regulation of GRP78, ATF4, GRP75, and TID-1 protein abundance. Maternal treatment with the mitochondria-targeted antioxidant MitoQ in hypoxic pregnancy further increased placental maternal blood space surface area and volume and restored activation of the ATF4 pathway, normalizing UPR and mitochondrial stress signaling mechanisms toward levels observed in normoxic pregnancy.

### Effects of Hypoxic Pregnancy on Placental Morphology and Fetal Biometry

In the rat, the placenta is fully developed by approximately day 14 of gestation.^63^ This means that in the present model of hypoxic pregnancy, the placenta developed under hypoxic conditions. In this study, it was demonstrated that the placenta adapts morphologically to early-onset hypoxia by increasing placental volume. Volumes of the decidua basalis, junctional zone, and labyrinthine zone were proportionally larger in hypoxic pregnancy, in association with expansion of the fetal capillary surface area within the labyrinthine zone. No changes were observed in the volume or surface area of maternal blood spaces or thickness of the placental interhaemel membrane. Similar beneficial changes in placental vascularization have been observed in the placentas of mice (13% oxygen, days 1 to 19^15^ and days 14 to 19^13^) and rats (11% oxygen, days 7 to 14^16^, ^17^) exposed to hypoxia from early to midpregnancy and in human pregnancy at high altitude.^56^, ^64^ The increase in fetal capillary blood surface area may represent a compensatory adaptation to increase or maintain placental transport capacity, thereby protecting fetal growth. By contrast, hypoxic pregnancy treated with MitoQ not only increased placental volume and fetal capillary surface area in the labyrinthine zone but also expanded maternal blood spaces. The thickness of the placental interhaemel membrane was not altered. The ability of MitoQ to enhance maternal blood perfusion of the hypoxic placenta may represent an additional protective mechanism to enhance the delivery of substrates for fetal growth. Accordingly, data in the present study also show that maternal treatment with MitoQ in hypoxic pregnancy also restored the impaired placental efficiency to control levels. Nitric oxide (NO) is important for the maintenance of umbilical blood flow; an increase in NO bioavailability can promote umbilical vasodilatation. The antioxidants melatonin and vitamin C can increase umbilical blood flow via NO-dependent mechanisms.^65^ MitoQ has been shown to improve endothelial function in aged mice^66^ and stroke-prone spontaneously hypertensive rats,^39^ by enhancing NO bioavailability. Substantial evidence suggests that endothelium-derived NO is a major mediator of angiogenesis.^67^ Taken together, these lines of evidence suggest that the enhanced volume of maternal blood spaces in the placenta of MitoQ-treated hypoxic pregnancies may be secondary to an increase in NO availability and NO-induced angiogenesis of uterine vessels that supply the labyrinthine zone.

### Effects of Hypoxic Pregnancy on Unfolded Protein Response and Cell Proliferation Signaling Mechanisms

There are three arms of the UPR signaling pathway, including PERK, ATF6, and inositol-requiring enzyme. Our previous publications have demonstrated only activation of the PERK–eukaryotic initiation factor 2α–ATF4 arm of the pathway in mice housed under hypoxic conditions,^15^ in human placentas from high altitude,^23^ and in trophoblast cells exposed to 1% O_2_.^23^ Therefore, the PERK arm of the UPR signaling pathway was studied. ATF4 expression is a known readout of the phosphorylation status of eukaryotic initiation factor 2α. We have previously reported activation of eukaryotic initiation factor 2α when tissue was collected 30 minutes after placental separation from the uterine wall.^68^ In comparison to the process of phosphorylation, which rapidly switches on and off, the expression of the *ATF4* gene and then translation into proteins take considerably longer and are less influenced by tissue collection and handling. Therefore, ATF4 was considered as a biomarker for ER stress in the present study. GRP78 protein abundance was shown to be increased in the placenta of hypoxic pregnancy, with or without MitoQ treatment. In addition, ATF4 protein abundance was significantly elevated in hypoxic pregnancy but restored to normoxic levels with MitoQ treatment. GRP78, an ER chaperone protein, plays a crucial role in the regulation of the ER dynamic equilibrium and guides misfolded proteins out of the ER and into the cytosol for degradation.^69^ PERK-ATF4 is a key UPR signaling mechanism in the adaptive response of cells to oxidants and increases in response to cellular stresses.^70^ Under hypoxic conditions, there is not only an increase in mitochondrial reactive oxygen species production but also a disruption of calcium homeostasis in the mitochondria, cytosol, and ER.^71^ Loss of calcium from the ER lumen, which leads to a perturbation in ER homeostasis, is one of the major triggers of the UPR.^72^ Therefore, the data suggest that early-onset hypoxic pregnancy up-regulates placental GRP78 in an attempt to reestablish ER homeostasis and resolve ER stress. On the other hand, activation of the PERK-ATF4 pathway may increase oxidative defense mechanisms by facilitating antioxidant enzyme expression.^73^ Indeed, this hypothesis is supported in the present study in hypoxic pregnancy supplemented by MitoQ. In this instance, the lack of up-regulation of ATF4 in response to increased placental GRP78 implies that exogenous MitoQ supplementation renders the activation of placental oxidative defense mechanisms unnecessary. Our data support previous studies in which GRPs have been shown to be induced by hypoxic conditions.^74^, ^75^, ^76^ Severe hypoxia or anoxia has been shown to activate ATF4.^77^, ^78^ Of interest, both GRP78 and AFT4 protein levels have been shown to be up-regulated in the placentas of women with either early- or late-onset preeclampsia.^79^, ^80^, ^81^

The AKT–mammalian target of rapamycin signaling pathway plays a crucial role in the regulation of placental size. AKT–mammalian target of rapamycin signaling has been shown to be up-regulated in pregnancies from obese women^82^ and down-regulated in placentas from growth-restricted pregnancies.^28^ In relation to hypoxic pregnancy, studies have shown both up-regulation and down-regulation of this pathway, in rodent and human pregnancies.^13^, ^15^, ^23^ In the present study, placental AKT and phosphorylated AKT (Thr308) protein expression remained unchanged, despite an increase in placental volume in hypoxic pregnancy. This suggests that other growth regulatory pathways may be involved, such as the mitogen-activated protein kinase.^83^

In the current study, there was no evidence of oxidative stress or lipid peroxidation in hypoxic placentas with or without MitoQ treatment. However, the immunostaining of the mitochondrial stress markers GRP75 and TID-1 was found to be increased in the placentas of hypoxic pregnancies but restored with MitoQ treatment. There is extensive evidence in the literature of studies, including our own, for the protection of mitochondrial function *in vivo* by MitoQ treatment in other tissues from various animal models of pathology, including the liver,^84^ heart,^85^ kidney,^86^ and vascular endothelial cells.^66^ Taken together, our data, therefore, demonstrate that hypoxia induces a low-grade ER and mitochondrial stress by activating the PERK–eukaryotic initiation factor 2α–ATF4 pathway; however, treatment of hypoxic pregnancy with MitoQ was effective in suppressing their activation.

### MitoQ Uptake during Pregnancy

In the current study, MitoQ was administered at a dose of 500 μmol/L in the dam's drinking water, from day 6 to day 20 of pregnancy. This equated to approximately 0.044 mg MitoQ/g per day. Liquid chromatography–tandem mass spectrometry results indicated that MitoQ uptake by the placenta and maternal liver was considerably greater than that of the fetal liver. The range of tissue concentrations of MitoQ in the placenta (approximately 105 pmol/g) and maternal liver (approximately 180 pmol/g) is comparable to concentrations that have been demonstrated to protect cells in culture from oxidative damage.^87^ Previous studies in which the same dose was administrated to mice in drinking water over several weeks demonstrated a rapid steady-state distribution of the compound in the heart, liver, kidneys, and skeletal muscle.^36^ During pregnancy, MitoQ uptake appears low in the fetus. This suggests that the potential benefit to the fetus of MitoQ supplementation at this dose during complicated pregnancy is via actions directly on the placenta. These findings are in keeping with the protective effects of MitoQ on fetal brain development, despite being bound to nanoparticles that prevented transfer of the antioxidant to the fetus.^49^

### Maternal Hematocrit and Food and Water Intake

Hypoxia-inducible factors orchestrate the classic physiological response to systemic hypoxia that results in increased erythropoietin levels and an increase in red blood production.^88^ MitoQ in hypoxic pregnancy did not prevent the increase in maternal hematocrit measured in untreated hypoxic pregnancy, suggesting that supplementation with MitoQ does not affect maternal oxygen sensing. In the present study, maternal food and water intake, as well as maternal weight, were transiently affected by maternal treatment with MitoQ in both normoxic and hypoxic pregnancy. This suggests that the pregnant rats possibly had to adapt to the taste of MitoQ. However, in human clinical trials with MitoQ administration, possible taste adversity has been satisfactorily resolved by formulating treatment via a tablet.^48^, ^89^

### Future Directions

There is growing evidence for the importance of addressing sex differences in the programing of disease by adverse prenatal conditions. The placentas from male offspring were studied because males appear more sensitive to altered oxygen and supply because of their higher rate of intrauterine growth, relative to females.^90^ In the present study, sex differences were controlled, but not addressed. Future studies should examine the sex-specific effects of hypoxic pregnancy, with or without antioxidant treatment, on placenta phenotype.

Although maternal antioxidant therapy was administered from the onset of chronic fetal hypoxia, which may limit translation to the clinic, the data provide proof of principle that mitochondria-targeted antioxidants may be beneficial in complicated pregnancy. Clinically, diagnosis of chronic fetal hypoxia would need to be established before the induction of maternal antioxidant treatment. Studies in chick embryos have reported that treatment of hypoxic incubations with agents that increase NO bioavailability or antioxidants, such sildenafil or melatonin, can protect against cardiovascular dysfunction in the offspring, even when therapy is started 12 days after the induction of chronic hypoxia.^91^, ^92^ The chick embryo may, therefore, prove a useful model to further assess human translational mitochondrial-targeted antioxidant therapies in pregnancies complicated by hypoxia.

## Conclusions

Early-onset hypoxic pregnancy in rodents induces morphologic adaptations in the placenta that offset increased placental UPR signaling, aiming to sustain fetal growth. Maternal treatment with the mitochondria-targeted antioxidant MitoQ in hypoxic pregnancy conferred protection against placental UPR activation and mitochondrial stress; and it further modified placental morphology by increasing the maternal blood spaces. The data suggest that mitochondria-targeted antioxidants may be beneficial in complicated pregnancies and minimize the detrimental effects on fetal development of reduced oxygen delivery via mechanisms protecting against activation of the placental UPR, thereby enhancing placental perfusion and efficiency.

## Supplemental Data

Data Profile
